# Supplementary material for: Early life factors associated with childhood trajectories of violence among the Birth to Twenty-Plus Cohort in Soweto, South Africa
Source: PLoS One. 2025 Nov 19;20(11):e0294207. doi: 10.1371/journal.pone.0294207 (PMC12629478; doi:10.1371/journal.pone.0294207)
Supplement: S1 Table — (DOCX) [file pone.0294207.s001.docx]

S1 Supplementary table 1: Proportions of physical and sexual violence victimization per age

|  | **Age (years)** | **Yes n (%)** | **No n (%)** | **Total** |
| --- | --- | --- | --- | --- |
| **Physical violence victimization** | | | | |
|  | 5 | 197 (14.0) | 1207 (86.0) | 1404 |
|  | 11 | 772 (56.7) | 589 (43.3) | 1361 |
|  | 15 | 1400 (71.8) | 550 (28.2) | 1950 |
|  | 18 | 717 (38.6) | 1142 (61.4) | 1859 |
| **Sexual violence victimization** | | | | |
|  | 5 | 11 (0.8) | 1383 (99.2) | 1394 |
|  | 11 | 49 (4.0) | 1167 (96.0) | 1216 |
|  | 15 | 325 (16.7) | 1624 (83.3) | 1949 |
|  | 18 | 528 (28.9) | 1302 (71.2) | 1830 |
