# Supplementary material for: Early life factors associated with childhood trajectories of violence among the Birth to Twenty-Plus Cohort in Soweto, South Africa
Source: PLoS One. 2025 Nov 19;20(11):e0294207. doi: 10.1371/journal.pone.0294207 (PMC12629478; doi:10.1371/journal.pone.0294207)
Supplement: S2 Table — (DOCX) [file pone.0294207.s002.docx]

S2 Supplementary table 2: Characteristics of excluded and included sample for physical violence victimization trajectory analyses

| **Variables** | **Total N (%)** | **Included n (%)** | **Excluded n (%)** | **P-values** |
| --- | --- | --- | --- | --- |
|  | 3269 | 2057 (62.9) | 1212 (37.1) |  |
| **Individual factors** | | | | |
| **Sex** | | | | 0.073 |
| Male | 1592 (48.7) | 977 (47.5) | 615 (50.7) |  |
| Female | 1677 (51.3) | 1080 (52.5) | 597 (49.3) |  |
| **Birthweight** | | | | 0.206 |
| Low birth weight (<2500 grams) | 350 (10.7) | 231 (11.2) | 119 (9.8) |  |
| Normal birthweight (≥ 2500 grams) | 2913 (89.2) | 1822 (88.8) | 1091 (90.2) |  |
| **Infant and child growth factors** | | | |  |
| Relative weight gain 0-2 years ^a^ | 1785 | -0.03 (0.99) | 0.11 (1.02) | 0.01 |
| Relative weight gain 2-5 years ^a^ | 1552 | 0.00 (1.03) | 0.02 (0.81) | 0.741 |
| Relative height gain 0-2 years ^a^ | 1786 | -0.04 (0.98) | 0.15 (1.04) | 0.001 |
| Relative height gain 2-5 years ^b^ | 1552 | -0.02 (-0.62; 0.62) | -0.10(-0.55; 0.49) | 0.671 |
| **Family level factors** | | | | |
| **Household socioeconomic status** | | | | <0.001 |
| Low | 996 (34.9) | 606 (32.4) | 390 (39.6) |  |
| Middle | 1401 (49.1) | 1003 (53.6) | 398 (40.5) |  |
| High | 459 (16.1) | 263 (14.1) | 196 (19.9) |  |
| **Household crowding** | | | | <0.001 |
| Yes | 1020 (38.6) | 777 (43.7) | 243 (28.0) |  |
| No | 1626 (61.5) | 1001 (56.3) | 625 (72.0) |  |
| **Maternal age** | | | | 0.141 |
| ≤ 24 years | 1493 (45.7) | 962 (46.8) | 531 (43.8) |  |
| 25 – 34 years | 1440 (44.1) | 879 (42.7) | 561 (46.3) |  |
| ≥ 35 years | 336 (10.3) | 216 (10.5) | 120 (9.9) |  |
| **Maternal Parity** | | | | 0.025 |
| 1 child | 1197 (36.6) | 783 (38.1) | 414 (34.2) |  |
| >1 child | 2072 (63.4) | 1274 (61.9) | 798 (65.8) |  |
| **Marital status** | | | | <0.001 |
| Married | 1413 (43.5) | 773 (37.8) | 640 (53.2) |  |
| Single | 1834 (56.5) | 1270 (62.2) | 564 (46.8) |  |
| **Maternal education status** | | | | <0.001 |
| Primary & below | 454 (15.5) | 232 (12.3) | 222 (21.3) |  |
| Secondary | 2148 (73.4) | 1491 (79.1) | 657 (63.0) |  |
| Post school training | 326 (11.1) | 162 (8.6) | 164 (15.7) |  |

| **Variables** | **Total N (%)** | **Included n (%)** | **Excluded n (%)** | **P-values** |
| --- | --- | --- | --- | --- |
| **Paternal education status** | | | | <0.001 |
| Primary & below | 220 (9.9) | 121 (8.3) | 99 (13.0) |  |
| Secondary | 1520 (68.6) | 1061 (72.9) | 459 (60.2) |  |
| Post-school training | 477 (21.5) | 273 (18.8) | 204 (26.8) |  |
| **Father present** | | | | 0.226 |
| Yes | 1966 (85.3) | 1568 (84.9) | 398 (87.1) |  |
| No | 339 (14.7) | 280 (15.2) | 59 (12.9) |  |
| **Maternal prior violence experience** | | | | 0.469 |
| Yes | 302 (18.9) | 184 (18.4) | 118 (19.8) |  |
| No | 1295 (81.1) | 818 (81.6) | 477 (80.2) |  |
| **Mode of delivery** | | | | 0.986 |
| Vaginal delivery | 1468 (88.2) | 1012 (88.2) | 456 (88.2) |  |
| Assisted delivery | 196 (11.8) | 135 (11.8) | 61 (11.8) |  |

^a^ Reported as means (standard deviation), ^b^ reported as medians (interquartile range)
