# Supplementary material for: Early life factors associated with childhood trajectories of violence among the Birth to Twenty-Plus Cohort in Soweto, South Africa
Source: PLoS One. 2025 Nov 19;20(11):e0294207. doi: 10.1371/journal.pone.0294207 (PMC12629478; doi:10.1371/journal.pone.0294207)
Supplement: S3 Table — (DOCX) [file pone.0294207.s003.docx]

S3 Supplementary table 3. Characteristics of included and excluded sample for sexual violence victimization trajectory analyses

| **Variables** | **Total N (%)** | **Included n (%)** | **Excluded n (%)** | **P-values** |
| --- | --- | --- | --- | --- |
|  | 3269 | 2051 (62.7) | 1218 (37.3) |  |
| **Individual level factors** | | | | |
| **Sex** | | | | 0.114 |
| Male | 1592 (48.7) | 977 (47.5) | 615 (50.5) |  |
| Female | 1677 (51.3) | 1074 (52.5) | 603 (49.5) |  |
| **Birthweight** | | | | 0.222 |
| Low birth weight (<2500 grams) | 350 (10.7) | 230 (11.2) | 120 (9.9) |  |
| Normal birthweight (≥ 2500 grams) | 2913 (89.2) | 1817 (88.8) | 1096 (90.1) |  |
| **Infant and child growth factors** | | | |  |
| Relative weight gain 0-2 years ^a^ | 1785 | -0.03 (0.99) | 0.11 (1.02) | 0.011 |
| Relative weight gain 2-5 years ^a^ | 1552 | -0.01 (1.03) | 0.03 (0.81) | 0.519 |
| Relative height gain 0-2 years ^a^ | 1786 | -0.04 (0.98) | 0.14 (1.04) | 0.001 |
| Relative height gain 2-5 years ^b^ | 1552 | -0.02 (-0.62; 0.62) | -0.12 (-0.55; 0.51) | 0.723 |
| **Family level factors** | | | | |
| **Household socioeconomic status** | | | | <0.001 |
| Low | 996 (34.9) | 602 (32.2) | 394 (40.0) |  |
| Middle | 1401 (49.1) | 1002 (53.6) | 399 (40.5) |  |
| High | 459 (16.1) | 266 (14.2) | 193 (19.6) |  |
| **Household crowding** | | | | <0.001 |
| Yes | 1020 (38.6) | 778 (43.8) | 242 (27.8) |  |
| No | 1626 (61.5) | 997 (56.2) | 629 (72.2) |  |
| **Maternal age** | | | | 0.115 |
| ≤ 24 years | 1493 (45.7) | 961 (46.9) | 532 (43.7) |  |
| 25 – 34 years | 1440 (44.1) | 875 (42.7) | 565 (46.4) |  |
| ≥ 35 years | 336 (10.3) | 215 (10.5) | 121 (9.9) |  |
| **Maternal Parity** | | | | 0.013 |
| 1 child | 1197 (36.6) | 784 (38.2) | 413 (33.9) |  |
| >1 child | 2072 (63.4) | 1267 (61.8) | 805 (66.1) |  |
| **Marital status** | | | | <0.001 |
| Married | 1413 (43.5) | 773 (37.9) | 640 (53.0) |  |
| Single | 1834 (56.5) | 1266 (62.1) | 568 (47.0) |  |
| **Maternal education status** | | | | <0.001 |
| Primary & below | 454 (15.5) | 233 (12.4) | 221 (21.2) |  |
| Secondary | 2148 (73.4) | 1490 (79.1) | 658 (63.0) |  |
| Post school training | 326 (11.1) | 161 (8.6) | 165 (15.8) |  |

| **Variables** | **Total N (%)** | **Included n (%)** | **Excluded n (%)** | **P-values** |
| --- | --- | --- | --- | --- |
| **Paternal education status** | | | | <0.001 |
| Primary & below | 220 (9.9) | 120 (8.3) | 100 (13.1) |  |
| Secondary | 1520 (68.6) | 1060 (73.0) | 460 (60.1) |  |
| Post-school training | 477 (21.5) | 272 (18.7) | 205 (26.8) |  |
| **Father prese** | | | | 0.196 |
| Yes | 1966 (85.3) | 1564 (84.8) | 402 (87.2) |  |
| No | 339 (14.7) | 280 (15.2) | 59 (12.8) |  |
| **Maternal prior violence experience** | | | | 0.520 |
| Yes | 302 (18.9) | 185 (18.4) | 117 (19.7) |  |
| No | 1295 (81.1) | 819 (81.6) | 476 (80.3) |  |
| **Mode of deliver** | | | | 0.921 |
| Vaginal delivery | 1468 (88.2) | 1006 (88.2) | 462 (88.3) |  |
| Assisted delivery | 196 (11.8) | 135 (11.8) | 61 (11.7) |  |

^a^ Reported as means (standard deviation), ^b^ reported as medians (interquartile range)
