# Supplementary material for: Early life factors associated with childhood trajectories of violence among the Birth to Twenty-Plus Cohort in Soweto, South Africa
Source: PLoS One. 2025 Nov 19;20(11):e0294207. doi: 10.1371/journal.pone.0294207 (PMC12629478; doi:10.1371/journal.pone.0294207)
Supplement: S4 Table — (DOCX) [file pone.0294207.s004.docx]

Supplementary table 4. Physical and sexual violence victimization trajectory model selection and adequacy

| **Number of groups** | **Polynomial order** | **BIC** | **Entropy** | **APP** | **Lowest assigned group membership (%)** |
| --- | --- | --- | --- | --- | --- |
| **Physical violence victimization trajectory models** | | | | | |
| 1 | 3 | -3917.55 | - | - | - |
| 2 | 3 3 | -3909.63 | 0.59 | 0.93; 0.70 | 20.47 |
| 3 | 3 3 3 | -3930.31 | 0.57 | 0.84; 0.56; 0.60 | 8.46 |
| **2** | **3 1** | **-3904.30** | **0.56** | **0.93; 0.75** | **34.86** |
| **Sexual violence victimization trajectory models** | | | | | |
| 1 | 2 | -2263.51 | - | - | - |
| 2 | 2 2 | -2271.08 | 0.44 | 0.71; 0.89 | 25.94 |
| 3 | 2 2 2 | -2282.35 | 0.66 | 0.71; 0.89; 0 | 0 |
| **2** | **2 3** | **-2275.49** | **0.57** | **0.84; 1.00** | **25.94** |

BIC - Bayesian information criterion, APP - Average posterior probabilities
