# Supplementary material for: Early life factors associated with childhood trajectories of violence among the Birth to Twenty-Plus Cohort in Soweto, South Africa
Source: PLoS One. 2025 Nov 19;20(11):e0294207. doi: 10.1371/journal.pone.0294207 (PMC12629478; doi:10.1371/journal.pone.0294207)
Supplement: S5 Text — (DOCX) [file pone.0294207.s005.docx]

# S5 Supplementary text. Sample Power calculation

Of the total 3,273 children recruited at birth, 2,057 and 2051 children followed up from birth to 18 years had data on physical and sexual violence victimization in at least two of the four-time (5, 11, 15 and 18) points of data collection, respectively. Stata version 17 was used to calculate power based on two sample proportions at 95% confidence interval.

Exposure variable selected for power calculation was household socioeconomic status. This variable was re-categorized from three categories (lower, middle and higher) to a binary variable comprising of low and high (average and high combined) household socioeconomic status. However, only 1,872 and 1,870 children had responses on household socioeconomic status at birth for physical and sexual violence victimization trajectory analyses, respectively. These samples will be used to represent the minimum sample size used for power calculation using the proportions described below;

For physical violence victimization analyses, the proportion of children from low and high socioeconomic status households were 65.0% (1,217) and 35% (655) respectively. 37.1% of children from households with low socioeconomic status and 29.9% of children from households with high socioeconomic status were assigned to the chronic increasing trajectory group.

For sexual violence victimization, 64.8% (1,212) of children resided in households with low socioeconomic status, while 35.2% (658) of the children resided in high socioeconomic households. 28% of children from households with low socioeconomic status and 22% of children from households with high socioeconomic status belonged to the late increasing sexual violence victimization trajectory group.

Using power two proportions command on stata and the above proportions, this study had 86.3% and 81.3% power to detect a statistically significant difference in physical and sexual violence trajectory group membership between children residing in households with low and high socioeconomic status, respectively.
