# Supplementary material for: Early life factors associated with childhood trajectories of violence among the Birth to Twenty-Plus Cohort in Soweto, South Africa
Source: PLoS One. 2025 Nov 19;20(11):e0294207. doi: 10.1371/journal.pone.0294207 (PMC12629478; doi:10.1371/journal.pone.0294207)
Supplement: Attachment — Submitted filename: Response to Reviewers.docx [file pone.0294207.s006.docx]

University of Witwatersrand

27 St Andrews Road, Parktown

Johannesburg, South Africa

19 August 2024

**RE: Revised manuscript submission – Early life factors associated with childhood trajectories of violence among the Birth to Twenty-Plus Cohort in Soweto, South Africa**

To: The Editorial team

The authors of the manuscript titled “Early life factors associated with childhood trajectories of violence among the Birth to Twenty-Plus Cohort in Soweto, South Africa,” appreciate the time and effort taken by the reviewers in evaluating our work. All the comments were carefully considered, and corresponding revisions were made to enhance the quality and clarity of the manuscript.

A detailed response to each reviewer’s comments, outlining how each comment has been addressed, is annexed in this letter. The authors kindly request your assistance in updating the revisions to the Financial Disclosure and Data Availability Statement in the online submission form on our behalf.

We believe the revisions have strengthened the manuscript, and we will be grateful if the revised version could be considered for publication in the PLOS ONE journal.

We look forward to your favorable response.

Sincerely,

Lilian Muchai

Corresponding author

[muchailily@gmail.com](mailto:muchailily@gmail.com)

Tel. +27 (81) 537 1075

**Response to reviewers**

| **Reviewer comment** | **Author response** | **Page #** |
| --- | --- | --- |
| **Journal requirements** | | |
| Please state what role the funders took in the study.  If the funders had no role, please state: "The funders had no role in study design, data collection and analysis, decision to publish, or preparation of the manuscript."  If this statement is not correct you must amend it as needed.  [The Bt20 Plus study is funded by the Wellcome Trust (UK), the Medical Research Council of South Africa, the University of Witwatersrand and supported by the DST - NRF Centre of Excellence in Human Development at the University of Witwatersrand, Johannesburg. The funders had no role in the study design, analysis, decision to publish or preparation of the manuscript. | Statement edited as below:  The Bt20 Plus study was funded by the Wellcome Trust (UK), the Medical Research Council of South Africa, the University of the Witwatersrand and supported by the DSI - NRF Centre of Excellence in Human Development at the University of Witwatersrand, Johannesburg. This study was funded by the Sexual Violence Research Initiative (SVRI) through a research grant (Childhood trajectories of violence and adult intimate partner violence: A gendered, life course approach to violence prevention). The funders had no role in the study design, data collection and analysis, decision to publish or preparation of the manuscript. | - |
| In the online submission form you indicate that your data is not available for proprietary reasons and have provided a contact point for accessing this data. Please note that your current contact point is a co-author on this manuscript. According to our Data Policy, the contact point must not be an author on the manuscript and must be an institutional contact, ideally not an individual. Please revise your data statement to a non-author institutional point of contact, such as a data access or ethics committee, and send this to us via return email. Please also include contact information for the third party organization, and please include the full citation of where the data can be found.  “The Birth to Twenty- Plus Cohort data underlying the results presented in the study canbe requested from the Birth to Thirty Executive Committee (https://bt30.org/data/).  Alternatively, the authors can be contacted for a copy of the analytic dataset.” | To be edited to the statement below.  The Birth to Twenty- Plus Cohort data can be requested from the Birth to Thirty Executive Committee: Prof. Linda Ritcher, the executive committee chair, [linda.richter@wits.ac.za](mailto:linda.richter@wits.ac.za). The first five years of cohort data are available online at bt30.org (<https://bt30.org/data/> ). | - |
| Please review your reference list to ensure that it is complete and correct. If you have cited papers that have been retracted, please include the rationale for doing so in the manuscript text, or remove these references and replace them with relevant current references. Any changes to the reference list should be mentioned in the rebuttal letter that accompanies your revised manuscript. If you need to cite a retracted article, indicate the article’s retracted status in the References list and also include a citation and full reference for the retraction notice. | Minimal changes have been made to the reference list, in response to comments from Reviewer one addressed below. No papers cited in this manuscript have been retracted. | 28 - 36 |
| The paper includes many additional English language errors. Editing by an English language expert is needed and would significantly improve the article. | Thank you for this suggestion, the manuscript has been edited thoroughly to meet these requirements. | - |
| **Reviewer 1** | |  |
| The authors use the appropriate scientific technical language, objective and pertinent to the theme, although it can be summarized, in order to allow a fluid and direct reading, especially in the descriptions of results. It should be noted that in these aspects, tables are important elements of information to the reader.  The authors employ diverse, pertinent references, but with more than 5 years for the most part. This aspect can weaken the discussions specifically on this topic in the work. | The authors have summarized the results and other sections of the manuscript. Revisions made to the manuscript have incorporated recent references where possible. | - |
| The method is described clearly and objectively in order to allow the reader to understand it and the possibility of replicability is guaranteed. The reviewer takes the liberty of suggesting to the authors the presence of a safe, infographic, which can summarize the flow of the process and allow the reader a better understanding of the sequence of steps. | Thank you for this suggestion. Figure 1, an infographic that outlines the study design and flow of the study process, has been included in the revised manuscript (line 111) | 5 |
| The results presented are compatible with the objectives explained and resulting from the defined methodology. Sometimes the reader is faced with an amount of information that can compromise his understanding, to which the reviewer once again takes the liberty of suggesting, a presentation with the use of topics that would lead to the results, thus minimizing any possibility of confusion of the data presented. | Sub-topics under the results section have been included for better clarity, thank you. | 11 - 22 |
| The authors present results in tables and graphs, but it should be noted that the tables do not have margins or lines. | The lines and margins in the original tables may have been removed in versions shared with reviewers. We have ensured that they are in place in this manuscript. As per journal requirements, the tables previously split were merged in the revised manuscript. | - |
| It is an important, current and relevant study in the context of child and adolescent care in developing countries. | The authors appreciate the time taken by the reviewer to provide their valuable input. | - |
| **Reviewer 2** | |  |
| I appreciate the opportunity to review this interesting manuscript. The focus of the study is well-defined, and the originality of the research is unquestionable as it differs from other literature. The study also shows adequate knowledge and understanding of relevant literature on the concept. The variables used in the study are well-defined. The discussion section is well written, with the authors making adequate references to other studies related to the outcome of their study. Additionally, the sentences are clearly expressed and readable. This is a well-written manuscript, and I look forward to seeing it in print | The authors appreciate the reviewer's comments. |  |
| **Reviewer 3** | |  |
| I would suggest avoiding the terms ‘developed’ and ‘developing’ when describing countries, and would recommend consulting the following reference for some helpful insights on terminology. | The authors have replaced the terms *developing and developed countries* with the use of geographical distinctions, as per the referenced publication guidance. | - |
| Line 109: I think ‘covering’ should be removed, or remove the brackets around the years. | This sentence has been revised by removing the word covering (line 108). | 5 |
| Lines 390-391: This sentence seems repetitive of what was discussed in the previous paragraph. I would suggest consolidating these to avoid repetition. | The two paragraphs have been consolidated (line 371 – 386). | 22 - 23 |
| Page 23, lines 409-412: The comment about perpetrators, while relevant to the discussion, seems out of place in this paragraph, given that the following paragraph returns to the discussion of the age at which sexual violence is more prevalent. | The authors are in agreement with the statement being out of place. Given that the line preceding this line offers support for findings of high sexual violence in adolescence, this statement on perpetration was removed to make the document more concise and focused on sexual violence victimization. | - |
| Page 24, last paragraph: Given the prevalence of stunting in South Africa, and its association (not only in SA) with poor developmental outcomes, I think some discussion about the relationship between stunting and other vulnerabilities associated with violence would be helpful. The comment about overweight/obesity being a risk factors for bullying and teasing in childhood needs to be linked to the overall discussion about violence in this context. | The revised manuscript has outlined better the role of early infant stunting on later outcomes and how these are associated with violence victimization (line 420 – 425). | 24 |
| The last sentence of this paragraph (on page 25) would fit better earlier in the paragraph. | The sentence has been placed earlier in the paragraph (line 443 – 444) | 25 |
| Page 26 lines 464-466 and 470-472: The research cited needs to be further expanded on to better link to the current study’s findings and context. These make sense to me, as someone working in the South African context, but may not make sense to an international reader unfamiliar with the context. | The revised manuscript has addressed how the current study findings are linked to the South African context (line 447 – 455). | 26 |
| The conclusion feels like it’s missing some recommendations for this context (and other similar contexts), based on the findings. It would be helpful to expand on the potential early target prevention areas mentioned. | Recommendations based on this study findings have been included in the revised manuscript (line 488 – 497). | 27 - 28 |
| Overall I found the manuscript to be clearly written, and my suggestions for improvement are relatively minor, and are mainly for the discussion, which I think needs some tightening up. | The reviewer's inputs and suggestions are highly appreciated. | - |
