# Supplementary material for: Early life factors associated with childhood trajectories of violence among the Birth to Twenty-Plus Cohort in Soweto, South Africa
Source: PLoS One. 2025 Nov 19;20(11):e0294207. doi: 10.1371/journal.pone.0294207 (PMC12629478; doi:10.1371/journal.pone.0294207)
Supplement: Attachment — Submitted filename: Response_to_Reviewers_auresp_2.docx [file pone.0294207.s007.docx]

University of Witwatersrand

27 St Andrews Road, Parktown

Johannesburg, South Africa

10 December 2024

**RE: Revised manuscript submission – Early life factors associated with childhood trajectories of violence among the Birth to Twenty-Plus Cohort in Soweto, South Africa**

To: The Editorial team

The authors of the manuscript titled “Early life factors associated with childhood trajectories of violence among the Birth to Twenty-Plus Cohort in Soweto, South Africa,” appreciate the time and effort taken by the reviewers in re-evaluating our work. All the comments were carefully considered, and corresponding revisions were made to enhance the quality and clarity of the manuscript.

A detailed response to each reviewer’s comments, outlining how each comment has been addressed, is annexed in this letter.

We believe the revisions have strengthened the manuscript, and we will be grateful if the revised version could be considered for publication in the PLOS ONE journal.

We look forward to your favorable response.

Sincerely,

Lilian Muchai

Corresponding author

[muchailily@gmail.com](mailto:muchailily@gmail.com)

Tel. +27 (81) 537 1075

**Response to reviewers**

| **Reviewer comment** | **Author response** | **Page #** |
| --- | --- | --- |
| **Journal requirements** | | |
| Please review your reference list to ensure that it is complete and correct. If you have cited papers that have been retracted, please include the rationale for doing so in the manuscript text, or remove these references and replace them with relevant current references. Any changes to the reference list should be mentioned in the rebuttal letter that accompanies your revised manuscript. If you need to cite a retracted article, indicate the article’s retracted status in the References list and also include a citation and full reference for the retraction notice. | Minimal changes have been made to the reference list, in response to improving the discussion section based on comments from Reviewer 4 addressed below. No papers cited in this manuscript have been retracted. | 29 - 37 |
| **Reviewer 4** | |  |
| The subject matter and content of this research is very interesting. A good amount of the mild hesitation I have about the manuscript is primarily of an overarching/general nature, but there are a few comments on specific suggestions as well | The authors appreciate the time taken by the reviewer to provide their valuable input. | - |
| Any findings with significance values of greater than 0.05 should likely not be termed "marginally significant" but rather "approaches significance" or "trend-level" or something to similar effect as from a term-of art standpoint a p>0.05 would not be "significant." | Thank you for this suggestion. The authors have replaced the term *marginally significant* with the term *approached significance*. | - |
| When there is an overwhelming amount of results data, it is helpful to provide organization for the reader in the Discussion section. Providing some structure in the Discussion about different categories/groupings of risk factors (by individual-level factor, family-level factor) or organizing the Discussion by victimization trajectory would provide a framework to provide clearer speculative explanations about potential data meaning in terms of interpretation as well as what it might implicate for next steps in research +/- intervention. | The authors have restructured the discussion section into separate paragraphs, each addressing a specific risk factor category and type of violence, to enhance clarity and provide a more detailed explanation of each area. | 22 - 29 |
| On page 26 of the manuscript there is a sentence that reads, "The spillover effects of post-Apartheid migration policies on family disruption, low marriage rates, alcohol and substance abuse and financial difficulties fathers face in providing for their families, have all contributed to increased rates of paternal absenteeism, non-residence or non-involvement in South African households [51]." While this general comment may have been highlighting a specific issue re: family structure factors, it speaks to some of the general impacts of societal stress that affect much of South African society--as the authors allude to at various points in the manuscript. If this is the case, it likely behooves the authors to do more to explain what protects youth from victimization group assignment. Said alternately: Are the risk factors *per se* predictive of assignment to victimization trajectories, or is it an interaction between a predictive factor and a wider social milieu? | Thank you for your thoughtful feedback. We appreciate your observation regarding the broader societal stressors impacting family structure and their potential implications for victimization group assignment. The authors provide additional context on how the residuals effects of post-apartheid policies have shaped family structures in South Africa. It is further suggested that, in South African context, attention should be given to the involvement of fathers or male figures in children’s lives, as research has shown this to be protective against violence victimization (line 445 – 451)  In addition, we have expanded our discussion to clarify the role of protective factors in mitigating the risk of victimization and highlight existing research on resilience mechanisms that shield youth from victimization despite exposure to adverse conditions (463 – 476) | 25 - 26  26 - 27 |
| The authors also highlight stunted development as something that is affects almost 30% of the overall population. After this study, an imagined intervention purportedly focused on children with stunted growth would most likely be a generalized primary prevention strategy rather than focused on a small population subset. There likely needs to be a clearer discussion not just of risk specificity of predictive factors, but also on whether the likely subsequent intervention strategies should be targeted vs. generalized across the entire youth population. Wrestling with the wider implications of this research and how it likely interacts with general social characteristics in the country would help to better contextualize how to interpret these results. | Thank you for this, we have amended that section to tackle the specificity of predictive factors and their implications for intervention design. Specifically, we consider how generalized primary prevention strategies might address widespread issues, while also acknowledging the potential benefits of targeted interventions for high-risk subpopulations. We emphasize the need for a hybrid approach that leverages both strategies to maximize effectiveness given the social and structural characteristics of the country. Additionally, we contextualize our findings by discussing how societal factors, such as systemic inequality and resource distribution, may shape the feasibility and impact of such interventions. These considerations aim to provide a more comprehensive interpretation of the results and their potential applications (line 515 – 526). | 29 |
| **Reviewer 5** | |  |
| I would like to thank you authors for addressing all previous comments so thoroughly. This is a well-written manuscript, with a robust analysis and sound conclusions. I have very minor comments:  Line 271: The figure 2 legend has been mislabelled.  Line 290: The figure 3 legend has been mislabelled. | The authors appreciate the reviewer's feedback and comments. Figure 2 (line 271) and 3 (line 290) legends have been labelled correctly. | 15, 16 |
