# Supplementary material for: Early life factors associated with childhood trajectories of violence among the Birth to Twenty-Plus Cohort in Soweto, South Africa
Source: PLoS One. 2025 Nov 19;20(11):e0294207. doi: 10.1371/journal.pone.0294207 (PMC12629478; doi:10.1371/journal.pone.0294207)
Supplement: Attachment — Submitted filename: Response_to_Reviewers_auresp_3.docx [file pone.0294207.s009.docx]

University of Witwatersrand

27 St Andrews Road, Parktown

Johannesburg, South Africa

18 August 2025

**RE: Revised manuscript submission – Early life factors associated with childhood trajectories of violence among the Birth to Twenty-Plus Cohort in Soweto, South Africa**

To: The Editorial team

The authors of the manuscript titled “Early life factors associated with childhood trajectories of violence among the Birth to Twenty-Plus Cohort in Soweto, South Africa,” appreciate the time and effort taken by the reviewers in re-evaluating our work. All the comments were carefully considered, and corresponding revisions were made to enhance the quality and clarity of the manuscript.

A detailed response to each reviewer’s comments, outlining how each comment has been addressed, is annexed in this letter.

We believe the revisions have strengthened the manuscript, and we will be grateful if the revised version could be considered for publication in the PLOS ONE journal.

We look forward to your favorable response.

Sincerely,

Lilian Muchai

Corresponding author

[muchailily@gmail.com](mailto:muchailily@gmail.com)

Tel. +27 (81) 537 1075

**Response to reviewers**

| **Reviewer comment** | **Author response** | **Page #** |
| --- | --- | --- |
| **Journal requirements** | | |
| I would like to ask you to carefully follow all the suggestions provided by the reviewers. In particular, I encourage you to strengthen the Discussion section by offering a more in-depth reflection on the limitations of the study. I also invite you to revise and expand the Conclusions, especially with regard to the broader implications of your findings. | Thank you. The authors have provided an in-depth critique of the limitations, in line with this and other reviewer suggestions, as well as expanded on and clearly marked the conclusion. |  |
| Please review your reference list to ensure that it is complete and correct. If you have cited papers that have been retracted, please include the rationale for doing so in the manuscript text, or remove these references and replace them with relevant current references. Any changes to the reference list should be mentioned in the rebuttal letter that accompanies your revised manuscript. | Minimal changes have been made to the reference list, in response to improving the introduction/ literature section based on comments from Reviewer 7 addressed below. No papers cited in this manuscript have been retracted. | 31 - 39 |
| **Reviewer 6** | |  |
| Given that this study uses secondary data from an existing longitudinal cohort, there are limitations in incorporating certain potentially relevant factors (such as maternal prior violence, mode of delivery, and infant growth) into the analyses due to high levels of missingness. While adjustments were made for variables that differed between included and excluded samples, selection bias cannot be fully ruled out. Notably, children included in the trajectory analyses were more likely to come from lower socioeconomic households with lower parental education, which may have influenced the observed associations. Additionally, the classification into trajectory groups was based on model fit indices (e.g., BIC, posterior probabilities, entropy), but the entropy values for both physical and sexual violence models were moderate (around 0.56–0.57), indicating some uncertainty in group assignment. This could attenuate the strength of associations between early life predictors and trajectory group membership. Lastly, while covariates were tested in multivariable models, the possibility of residual confounding remains, particularly due to the exclusion of relevant variables and potential multicollinearity among socioeconomic indicators. | The authors appreciate this comment. The possibility of selection bias due to the use of complete case analysis has been acknowledged in the revised manuscript (line 483 – 487).    The issue of residual confounding is also relevant, as most research cannot fully account for all risk factors associated with the outcome; this has been explicitly noted as a study limitation (Line 480 – 483).  Although multiple fit criteria were used to identify the best-fitting trajectory model, the moderate entropy values suggest potential limitations in classification accuracy, which may have influenced the observed associations. This point has been incorporated into the limitations section. Consistent with prior research, the authors have recommended that future studies compare results across different clustering methods and use a broader set of model adequacy indices to strengthen confidence in trajectory classification validity (line 499 – 508).  Finally, while multicollinearity is possible between variables related to socioeconomic status, diagnostic checks in the final multivariable model yielded low average variance inflation factor (VIF) values, indicating no evidence of problematic multicollinearity among the included variables. | 27 - 28 |
| While the limitations section addresses some key methodological concerns, it would benefit from additional clarification. Specifically, the use of secondary data limits control over the timing of measurements and the availability of certain variables, which should be more explicitly acknowledged. It is also important to note that the approach to missing data could introduce selection bias, and I recommend that future studies conduct sensitivity analyses to assess the robustness of trajectory groupings. Furthermore, although model diagnostics were conducted, residual confounding remains a possibility and should be highlighted more explicitly. | Thank you for this feedback, the authors have revised the limitation section to provide more clarity on the limitations associated with the use of secondary data (line 477 – 483).  Comment raised on residual confounding have been addressed in our response above | 27 |
| Regarding the conclusion, while it does reflect the main findings of the study, it would benefit from greater clarity and structure. Specifically, it would be helpful to more clearly distinguish between the patterns and associated factors of physical versus sexual violence victimization. Additionally, I suggest emphasizing the novelty of using trajectory modeling in a longitudinal cohort more strongly. The limitations section currently occupies a disproportionate amount of space and could be summarized more succinctly to maintain focus on the study’s contributions. Finally, the conclusion would be more impactful with a concise summary statement that clearly articulates the implications for future research and intervention strategies. | Thank you for these suggestions, the conclusion has been reworked to more clearly distinguish between the two trajectories, as well as to highlight the methodological novelty and provide a clear and concise summary of the implications (line 523 – 558).    We have tried to balance the length of the limitations section with requests for expanded explanations from other reviewers. | 29 - 30 |
| In the Methods section, I recommend providing a clearer distinction between the two-step analytical process—presenting univariable logistic regressions first, followed by multivariable modeling. Statistical terminology should also be refined for conceptual accuracy. For example, the term "correlates" (or "correlation") should be replaced with "associations," as the analyses assess relationships rather than statistical correlations. It would also be helpful to clarify that the variables included in the multivariable model were selected based on prior literature and data availability. Additionally, post-regression diagnostics should be described more explicitly, including a note that a test for multicollinearity was conducted to assess interdependence among explanatory variables. Finally, some minor edits are needed to improve clarity, precision, and consistency throughout the manuscript. | Thank you for this suggestion. The authors have revised the methodology section to enhance clarity regarding the progression from univariable to multivariable modelling (line 203 – 209)  Post-regression diagnostics are now described more explicitly (line 211 – 215), and mention of the term ‘correlates/ correlated’ substituted with the appropriate statistical terminologies. | 10 |
| **Reviewer 7** | |  |
| Lines - 117-124: how many participants were excluded from the study? how many participants were you left with? additionally, you could make it a bit clearer what distinguishes the inclusion/exclusion groups. i THINK it’s those who did vs. did not report experiencing violence, but making that more explicit would be helpful. perhaps the language of inclusion/exclusion makes it a bit confusing, since I’m assuming the “exclusion” category was removed from your analysis altogether, which seems not to be the case in your results (e.g., line 237). | Line 119-122 was to provide a justification to why only the four years (5,11, 15 and 18) out of the total 19 waves of data collection were used. This was based on availability of sufficient data on violence and only time point where physical and sexual data was collected separately were included. This has been revised to provide a clear and concise statement.  Line 229 - 231 shows that from the 4 included time points, only participants with data in 2 out of the 4 time points were included. We have also added the numbers of those excluded from the analysis (line 234 – 236). | 6  11 |
| Lines 151-152: It's not fully clear from the literature review/the method section why these are the risk factors of interest. You say “selected based on a priori knowledge and literature,” and I think this literature should be elucidated in your introduction/literature review. | Lines 74–87 have been revised to justify the focus on early life factors grounded in the developmental origins of health and disease (DOHaD) framework. The section also summarizes evidence from both longitudinal and cross-sectional studies to demonstrate the associations between each selected covariate and childhood violence victimization, thereby supporting their inclusion in the analysis. The reliance on secondary data restricted the scope of covariates to those available within the dataset, and this constraint has been explicitly acknowledged as a study limitation." | 4 |
| Line 231: You write, “had data on physical and sexual violence victimization," which is a bit unclear. Throughout the manuscript, you use this language of children “with data” or “having data” on violence throughout your paper. I’d love to see a more active description, such as “children who reported experiencing violence.” I think it would increase clarity. | Thank you for this suggestion. In the manuscript, references to ‘had /with data on violence’ has been revised to ‘children who reported any or no experiences of violence.’" to still accommodate for those children who report on the absence of violence. |  |
| Lines - 296-304: it would be helpful if you included in-text the logistic regression statistics, instead of only saying that you can find the results in tables 2 and 3. | Thank you for this feedback. This section provides a summary introduction to the results on early life factors associated with both physical and sexual violence victimization. To avoid repetition, detailed logistic regression statistics are presented separately for each violence type in the sections below. The authors have revised the summary introduction to enhance clarity and conciseness (line 297 – 304). | 16 - 17 |
